# Plasma Linoleate Diols Are Potential Biomarkers for Severe COVID-19 Infections

**DOI:** 10.3389/fphys.2021.663869

**Published:** 2021-04-01

**Authors:** Cindy B. McReynolds, Irene Cortes-Puch, Resmi Ravindran, Imran H. Khan, Bruce G. Hammock, Pei-an Betty Shih, Bruce D. Hammock, Jun Yang

**Affiliations:** ^1^Department of Entomology and Nematology, University of California, Davis, Davis, CA, United States; ^2^EicOsis Human Health Inc., Subsidiary of EicOsis LLC, Davis, CA, United States; ^3^Division of Pulmonary, Critical Care, and Sleep Medicine, Department of Internal Medicine, University of California, Davis, Davis, CA, United States; ^4^Department of Pathology and Laboratory Medicine, University of California, Davis, Davis, CA, United States; ^5^Department of Anatomy, Physiology, and Cell Biology, University of California, Davis, Davis, CA, United States; ^6^Department of Psychiatry, University of California, San Diego, San Diego, CA, United States; ^7^UCD Comprehensive Cancer Center, University of California, Davis, Davis, CA, United States

**Keywords:** linoleate diol, lipid mediators, COVID-19, inflammation, leukotoxin, EpOME, DiHOME, ARDS

## Abstract

Polyunsaturated fatty acids are metabolized into regulatory lipids important for initiating inflammatory responses in the event of disease or injury and for signaling the resolution of inflammation and return to homeostasis. The epoxides of linoleic acid (leukotoxins) regulate skin barrier function, perivascular and alveolar permeability and have been associated with poor outcomes in burn patients and in sepsis. It was later reported that blocking metabolism of leukotoxins into the vicinal diols ameliorated the deleterious effects of leukotoxins, suggesting that the leukotoxin diols are contributing to the toxicity. During quantitative profiling of fatty acid chemical mediators (eicosanoids) in COVID-19 patients, we found increases in the regioisomeric leukotoxin diols in plasma samples of hospitalized patients suffering from severe pulmonary involvement. In rodents these leukotoxin diols cause dramatic vascular permeability and are associated with acute adult respiratory like symptoms. Thus, pathways involved in the biosynthesis and degradation of these regulatory lipids should be investigated in larger biomarker studies to determine their significance in COVID-19 disease. In addition, incorporating diols in plasma multi-omics of patients could illuminate the COVID-19 pathological signature along with other lipid mediators and blood chemistry.

## Introduction

The pandemic coronavirus disease 2019 (COVID-19), caused by severe acute respiratory syndrome coronavirus 2 (SARS-CoV-2), initiates an aberrant immunological response resulting in a wide range of disease severities ranging from asymptomatic cases to severe cases with rapid progression to acute respiratory distress syndrome (ARDS) and death (Arentz et al., 2020; Du et al., 2020). Patients with severe COVID-19 show evidence of hyperinflammation with increased release of inflammatory cytokines (Pedersen and Ho, 2020). The role of a cytokine release syndrome, or cytokine storm, in COVID-19 has drawn much attention (Mehta et al., 2020). However, recent reports demonstrate that, although pro-inflammatory cytokine levels are elevated in severe COVID-19 patients, they are lower than levels usually observed in non-COVID ARDS, suggesting additional factors lead to severe outcomes in some patients (Sinha et al., 2020).

One of the key pathways regulating the immune response to infections is the release of regulatory lipid mediators that have dual functions of driving inflammation [e.g., prostaglandins (PGE2)] or promoting resolution of inflammation and return to homeostasis [e.g., long chain epoxy fatty acids (EpFAs)] (Dennis and Norris, 2015; Hammock et al., 2020). Recent data indicate a role of dysregulated lipid profiles in COVID-19 and identified cytochrome P450 (CYP) metabolites of polyunsaturated fatty acids (PUFA) as potential biomarkers of disease severity (Hammock et al., 2020; Schwarz et al., 2020).

**FIGURE 1:**
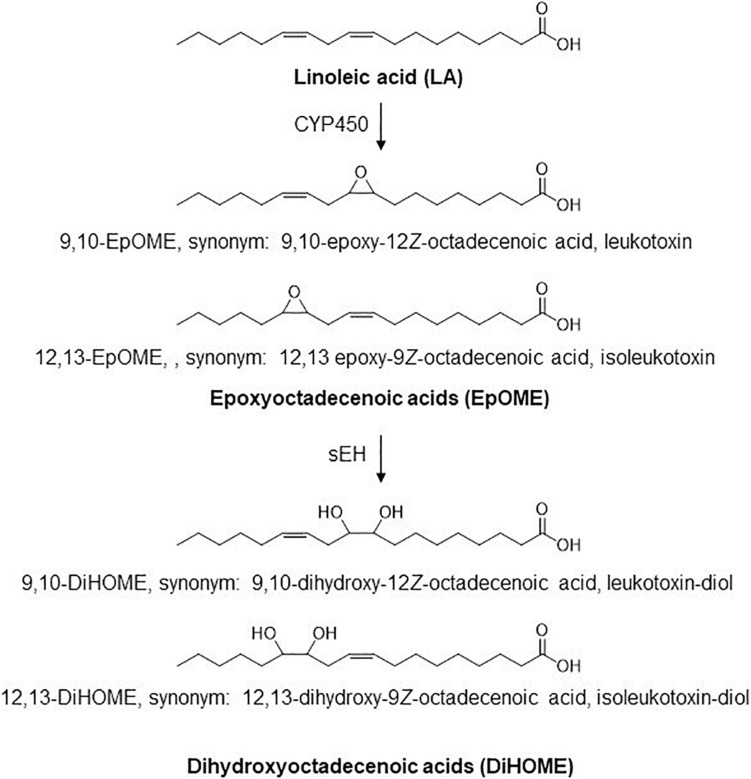
Structure of LA, EpOmE, and DiHOME.

Linoleic acid (18:2n6, LA) is the primary source of essential long chain n-6 PUFAs. CYP450 enzymes act on linoleate directly to generate linoleic epoxides (epoxyoctadecenoic acids, EpOMEs), which are further metabolized by soluble epoxide hydrolase (sEH) to their corresponding leukotoxin diols (dihydroxyoctadecenoic acids, or DiHOMEs; Figure 1). These LA metabolites regulate vascular permeability and stimulate neutrophil chemotaxis (Hildreth et al., 2020). The epoxides were originally termed leukotoxins because of their suspected cytotoxic effects and implications in advancing acute and chronic inflammatory diseases and in the pathophysiology of ARDS (Sugiyama et al., 1987; Zheng et al., 2001). The deleterious effects of LA metabolites were originally attributed to EpOMEs. It was later discovered that the toxicities attributed to leukotoxins were in fact driven by leukotoxin diols or DiHOMEs, and blocking their formation would alleviate toxicities previously associated with leukotoxin (Moghaddam et al., 1997). Despite its potential role in advancing ARDS, the role of these LA metabolites in the pathophysiology of COVID-19 has not been evaluated to date.

In this pilot study, five sequential day plasma samples from six patients with COVID-19 were profiled for lipidomic changes in COVID-19 disease compared to healthy controls. Results indicate that in addition to expected increases in inflammatory PGE2 and leukotrienes, 12,13 DiHOME and 9,10 DiHOME concentrations are significantly higher in COVID-19 patients compared to healthy controls. This is one of the first studies to focus on oxylipin chemical mediators in COVID-19 disease.

## Methods

This is a retrospective study using prospectively collected plasma samples and clinical/phenotype data. For oxylipin analysis, heparinized plasma was collected from six patients with laboratory-confirmed SARS-CoV-2 infection and admitted to the University of California Davis Medical Center in Sacramento, CA and 44 samples from healthy controls chosen from a recently completed clinical study. For comparison of cytokines, 75 plasma samples from healthy volunteers was obtained from the California Central Valley Delta Blood Bank (Stockton, CA, United States) prior to the COVID-19 pandemic. The methods used for blood collection, plasma processing, use of anti-coagulants/antioxidant/preservatives, and flash-freeze protocol were well-matched between case and control groups. The UC Davis and UC San Diego Institutional Review Boards have approved the use of anonymized biospecimens for this study.

### Lipid Mediator Profiling

Plasma (200 μL) samples were aliquoted to a cocktail solution including 600 μL of methanol with 10 μL of 500 nM of surrogate solution including 9 isotope-labeled oxylipins (d4 PGF1a, d4 PGE2, d4 TXB2, d4 LTB4, d6 20 HETE, d11 14,15 DiHETrE, d8 9 HODE, d8 5 HETE, and d11 11,12 EpETrE). Before the extraction, the samples were vortexed and centrifuged at 3,000 rpm in a biosafety hood. The supernatants were then loaded on prewashed SPE cartridges and washed with two column volumes of 5% MeOH solution before elution by 0.5 mL of MeOH and 1.5 mL of ethyl acetate. The eluents were dried under vacuum using the Nutec MaxiVac vacuum concentrator (Farmingdale, NY, United States) before reconstitution with 50 μL of 100 nM CUDA solution in methanol. Then, the extracted samples were analyzed using the UPLC/MS/MS system [Waters Acquity UPLC (Milford, MA, United States)] hyphenated to AB Sciex 6,500 + QTrap system (Redwood City, CA, United States). The detailed parameters for the UPLC/MS/MS method were described previously (Yang et al., 2009, 2019).

### Cytokine Multiplex

Plasma cytokines were measured using a multiplex magnetic bead-based cytokine detection kit purchased from Bio-Rad (12007283). Cytokines were measured according to manufacturer’s instructions. Data are provided in the Supplementary Material.

### Statistical Analysis

To test for differences between the COVID-19 and the control group cytokine levels, cytokine levels were log_10_ transformed to fit a normal distribution and analyzed in Graphpad Prism (version 8.4.3) using the Wilcoxon rank-sum test with COVID positive and negative status as the main effect.

Lipid mediator results were analyzed using MetaboAnalyst^1^ and scaled using autoscaling before analysis. Multiple data sets described below were integrated to prioritize the oxylipins as possible biomarkers contributing to the severity of COVID. Oxylipins were analyzed by multiple independent *t*-tests using patient vs. control as the variable and the two-stage step-up method of Benjamini, Krieger and Yekutieli to determine a false discovery rate (Benjamini et al., 2006) to generate the volcano plot.

The lipid mediators were then ranked by their effect sizes (i.e., the fold-difference between mean analyte concentration in each group). The analytes with the largest effect sizes were further evaluated by random effect ANOVA models. We minimized type 1 errors by testing for between-group differences among the analytes with the largest effect sizes and to improve the likelihood of identifying analytes that showed best potential to seve as biomarkers of disease severity. Each analyte with an effect size above 8 (i.e., analyte concentrations >8-fold different) was used as a response variable. Random effect ANOVAs were run with “patient” as a random effect to account for the multiple measurements from the same patient, and the fixed effect was “group” (i.e., COVID positive or control). The log_10_-transformation of the analyte concentrations was applied. The analysis was done in JMP Pro Version15.

## Results

Demographics of patient samples are represented in Table 1. Seventy-seven lipid mediators were detected from all the patients’ samples (oxylipin concentrations are available online: https://datadryad.org/stash/share/jYEfmOzrIkShoUT1fmMsT-lEl_dIbI7952I5EQ2kMk4). Levels of multiple key pro-inflammatory cytokines and chemokines were significantly higher in patients with COVID-19 than in healthy controls (Supplementary Dataset 1), confirming the activation of the immune response against the virus. Overall, increases were moderate and consistent with those reported in the literature (Mehta et al., 2020).

**TABLE 1:** Clinical characteristics of Sars-Cov-2 patients^1^.

Clinical ID/ #Patient ID^2^	Age	Covid19-symptoms						Onset (d)	Admission	Hospital stay (d)	Airway procedures performed	COVID-19 treatment
RIB00020 #1	<65	SOB^3^	FLS		Dyspnea			5–8		4	None	
RIB00019 #2	<65	SOB^3^	FLS	Fever	Dyspnea	Chest pain	Muscle pain	9–13		13	Supplemental oxygen	Remdesivir
RIB00012 #3	>65	SOB^4^		Fever	Dyspnea on exertion		Hypoxemia	5–8		16	Supplemental oxygen	
RIB00016 #4	<65	SOB^4^						9–13	AHRF/Pnu	11	Endotracheal intubation	
RIB00001 #5	<65		FLS					Several	ARDS/Pnu	26	Endotracheal intubation	Remdesivir
RIB00004 #6	>65							5–8	ARDS/Pnu	54	Endotracheal intubation	Sarilumab

*^1^All patients were above 45 years of age and had a cough upon admission. Clinical ID/Patient ID^2^ (patients were assigned a clinical ID at the hospital. For simplification, they were reassigned a number from 1–6. SOB^3^ (shortness of breath associate with other respiratory illness); SOB^4^ (shortness of breath); FLS (flu-like symptoms); AHRF (Acute Hypoxic Respiratory Failure); ARDS (Acute Respiratory Disease); and Pnu (Pneumonia).*

**FIGURE 2:**
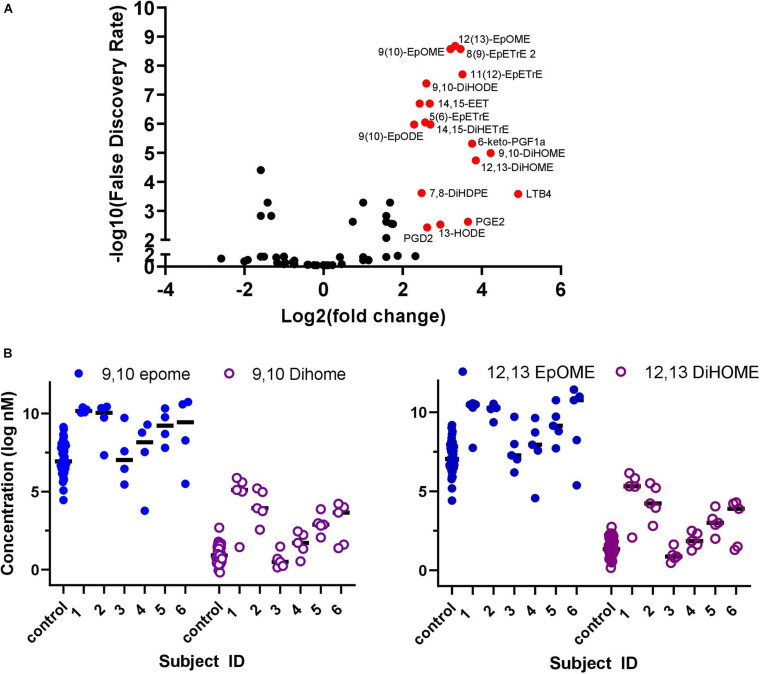
Plasma collected once from healthy COVID-19 negative controls (*n* = 44) and over five sequential days from hospitalized COVID-19 positive patients (*n* = 6). **(A)** Volcano plot of oxylipins analyzed in COVID-19 patients compared to healthy controls. Red dots identify metabolites that had a >4 fold-change and false discovery rate (*p* < 0.01), 18 compounds in total. To generate the Volcano plot, data from each COVID-19 patient was averaged over the 5-days before averaging as a group and comparing to the average of all control subject data. **(B)** Plasma concentration of EpOME and DiHOME in five sequential samples collected from six hospitalized COVID-19 positive patients and control samples collected separately from healthy volunteers (*n* = 44). Data from individual days is represented for each COVID patients and for each individual healthy control. The ratio of EpOME:DiHOME, excluding patient #3 who had low levels of both EpOMES and DiHOMES, was higher in control samples vs. COVID-19 patients on day 1 and steadily increased over time in COVID-19 patients. Overall ratios in COVID-19 patients were 30% lower than healthy controls indicating that DiHOMES increased in greater amounts compared to EpOME (graphical representation of raw data is included in Supplementary Figure 1).

A volcano plot analysis was performed to evaluate the differences in lipidomic profile between COVID-19 patients and healthy controls (Figure 2A). The analysis identified 18 differential lipid mediators with statistically significant differences (*p* < 0.01) with more than four-fold change between groups.

Oxylipins were ranked according to effect size (Table 2) between COVID-19 patients and controls. The 9,10 and 12,13 DiHOME metabolites had the biggest effect size (17.94 and 14.12, respectively), followed by PGE2 (12.55). As expected, the epoxides of arachidonic acid (AA) and linolenic acid also increased compared to healthy controls presumably due to biosynthesis and systemic release of free fatty acids from membranes in response to inflammation. The epoxides and diols of the omega-3 fatty acids, eicosapentaenoic acid (EPA) and docosahexaenoic acid (DHA), did not show any increases (effect size < 7).

**TABLE 2:** Effect size (mean fold-difference between COVID-positive and control) of EpFA, diols, and oxylipins with greater than 8-fold difference (*p < 0.0001).

Effect size of oxylipins compared to healthy controls			
	Effect size		Effect size
**LA metabolites**			
9(10)-EpOME	9.23*	9,10-DiHOME	17.94*
12(13)-EpOME	10.05*	12,13-DiHOME	14.12*
**ALA metabolites**			
9(10)-EpODE	4.90	9,10-DiHODE	7.36
12(13)-EpODE	3.21	12,13-DiHODE	0.88
15(16)-EpODE	5.39	15,16-DiHODE	0.37
**AA metabolites**			
5(6)-EpETrE	5.93	5,6-DiHETrE	2.98
8(9)-EpETrE	11.01*	8,9-DiHETrE	1.90
11(12)-EpETrE	11.41*	11,12-DiHETrE	2.07
PGE2	12.55		
**DHA metabolites**			
		4,5-DiHDPE	0.60
7(8)-EpDPE	1.04	7,8-DiHDPE	5.94
10(11)-EpDPE	0.88	10,11-DiHDPE	0.76
13(14)-EpDPE	0.75	13,14-DiHDPE	0.61
16(17)-EpDPE	0.74	16,17-DiHDPE	0.81
19(20)-EpDPE	3.28	19,20-DiHDPE	0.38
**EPA metabolites**			
8(9)-EpETE	0.95	8,9-DiHETE	0.93
11(12)-EpETE	1.27	11,12-DiHETE	0.95
14(15)-EpETE	1.03	14,15-DiHETE	0.87
17(18)-EpETE	1.35	17,18-DiHETE	0.43

*Increased EpFA from the most abundant dietary fatty acids (AA and LA) is expected due to release from cellular membranes in response to inflammation. AA epoxides, EpETrE or EETs are anti-inflammatory compounds, but their low concentration and rapid conversion by the sEH is thought to limit their efficacy.*

Figure 2B demonstrates that changes in the DiHOME concentrations had a more prominent effect in separating patients and controls compared to the EpOMEs. The EpOME/DiHOME ratios also demonstrated case-status predictive effects (Supplementary Figure 2B). The prostaglandins, PGE2 and PGD2, as well as related cyclooxygenase (COX) metabolites had large effect sizes but surprisingly were low in concentration in patients with evidence of elevated cytokines. It is worth noting that the large effect size of prostaglandin resulted from a single patient (see online dataset for individual data). Similarly, leukotriene B4 (LTB4, leukocyte aggregating factor) level was surprisingly low for patients with high level of inflammation, marked by elevated cytokines. This finding was also largely driven by the effect of one single patient.

## Discussion

It is clear that the two regioisomeric linoleic acid diols (DiHOMES) had highly elevated concentrations in these COVID-19 positive patients, as did their precursor epoxides (EpOMEs; Table 2). Previous studies show that high levels of the epoxide and diol metabolites of linoleic acid are mitochondrial toxins, stimulate vascular permeability and that injection of either metabolite into mice leads to pulmonary edema and inflammation reminiscent of ARDS (Greene and Hammock, 1999; Zheng et al., 2001). However, if inhibitors of the sEH are administered, the edema from EpOMEs is blocked but not that from the DiHOMEs (Moghaddam et al., 1997), suggesting that DiHOMEs play a role in lung disease and possibly a role in the pathophysiology of COVID-19. In contrast, the EET regioisomers and epoxides of other long chain PUFA are under scrutiny as inflammation resolving mediators. Their concentrations are quite low, and the sEH is thought to be largely responsible for converting the biologically active epoxides to their corresponding diols, thereby reducing their inflammation resolving potency. The online dataset represented by the volcano plot in Figure 2A and the effect sizes in Table 2 do not indicate that these compounds are associated with severe COVID, although increasing epoxides from arachidonic acid (ARA), EPA, and DHA to yield the EET, EEQ, and EDP regioisomers would be predicted to help resolve inflammation (Kodani and Hammock, 2015).

The levels of ARA diols from the corresponding EET epoxides as well as the epoxides and diols of omega-3 fatty acids were low in most subjects with relatively small differences between the COVID positive and control groups. A caution is that the data on omega-3 metabolites in human subjects can be hard to quantify in part because the average dietary levels of omega-3 fatty acids are low. Fatty acid composition, including omega-3 fatty acids, can become quite high due to supplementation. For example, the omega-6 fatty acid LA was once a relatively rare dietary lipid in our evolutionary history but is now a major dietary lipid in the western diet (Deol et al., 2017). In many western diets the levels of linoleate are far higher than that anticipated from even recent evolutionary history. As an example, the blood levels of the EpOMEs we report here in COVID-19 patients are approximately 10 × higher than levels found in ICU-admitted burn patients (Kosaka et al., 1994). Thus, the high levels of linoleate substrate would be expected to compete with long chain PUFA thus reducing inflammation-resolving epoxides, such as the EET_*S*_, EEQs and EDPs, and increasing linoleate epoxides or leukotoxins as shown by our data. Although AA and longer chain omega-3 fatty acid epoxides are better substrates for the sEH, the leukotoxins are still excellent substrates (Morisseau et al., 2010). sEH action on leukotoxins leads to metabolic products that are cytotoxic, proinflammatory, and cause extensive perivascular and alveolar edema reminiscent of ARDS in mice (Greene and Hammock, 1999; Zheng et al., 2001).

An obvious question remains as to why cells produce a pro-inflammatory metabolite which increases vascular permeability during COVID infection. A possible answer comes from an inspection of the AA cascade where the largely (but not exclusively) pro-inflammatory COX and lipoxygenase (LOX) pathways are countered by the more recently discovered and largely anti-inflammatory pathway termed the cytochrome CYP450 pathway (Kodani and Hammock, 2015). During inflammation, PUFA are released from cell membranes and are metabolized into epoxides thought to resolve inflammation; however, this process is often dysregulated in patients with severe disease. Specifically, while cytochrome P450 metabolism of PUFA forms mostly anti-inflammatory and inflammation-resolving fatty acid epoxides such as EETs, EDPs and EEQs (from AA, EPA, and DHA, respectively), metabolites from LA and other omega-6 PUFAs generated by other enzymes such as COX and LOX form mostly pro-inflammatory compounds. As shown in our results, the COX-generated prostaglandins (e.g., PGE2) and LOX-generated leukotrienes (LTB4) were increased as part of the inflammatory response during COVID-19. The EpFA resolve effects of these inflammatory eicosanoids directly through downregulation of inflammation, and indirectly by stimulating the production of specific proresolving mediators (SPMs). However, the sEH enzyme is up-regulated during inflammation, resulting in conversion of beneficial compounds into inactive or even pro-inflammatory diols. Under normal conditions, CYP450 oxidation of PUFA into EpFA is tightly regulated and occurs at a slower rate than the hydrolysis of EpFA into diols. EpFA are often stored in lipid membranes and are thought to be released during inflammation; however, the rapid conversion by sEH during inflammation limits their concentration *in vivo*.

The high abundance of linoleate as a substrate, coupled with the increased biosynthesis of anti-inflammatory EpFA during severe coronavirus infections and the induction of sEH in an inflammatory state (Kodani and Hammock, 2015) may explain the increased rate of synthesis and concentration of leukotoxin diols observed in COVID-19 patients in our study. This finding raises the possibility that amelioration of COVID-19 symptoms may be achieved in part by reduction of omega-6-rich diet, or an enhanced omega-3 fatty acid intake in patients hospitalized for COVID-19. Linoleate at quite low levels is an essential fatty acid for maintenance of skin barrier function, yet an early study (1958) showed that even with no dietary fat intake, 2% of energy from linoleate was enough to maintain skin barrier function (Hansen et al., 1958). Therefore, reducing linoleate intake or substituting it with “anti-inflammatory” lipids such as n-3 rich fish oil is unlikely to have a deleterious effect on the long-term health. Indeed, this approach is currently being evaluated through intravenous omega-3 administration in COVID-19 hospitalized patients in the COVID-Omega-F Trial (Arnardottir et al., 2021). Further benefits from reducing omega-6 fatty acid intake germane to the COVID-Omega-F Trial show increased bioavailability of omega-3 fatty acids with reduced LA consumption (Taha et al., 2014). Particularly the “omega” olefins of EPA and DHA are good substrates for epoxidation by relevant cytochrome P450s (Arnold et al., 2010). Thus, large infusions of omega-3 fatty acids would be predicted to reduce the biosynthesis of the omega-6 EpOMEs by substrate competition. A second prediction is that infusion of omega-3-fatty acids would not lead to a significant increase in COX products because of the substrate preferences of the COXs. On the other hand, the anti-inflammatory P450 products are expected to be increased. These EEQ and EDP epoxides also are excellent substrates for the sEH (Morisseau et al., 2010) and by competition should reduce the hydration of non-cytotoxic EpOMEs to the DiHOMEs (cytotoxic leukotoxin diols). A reduction in linoleate metabolites could partially explain the difference that omega-3 supplementation had in ARDS related mortality (Langlois et al., 2019). Given the evidence of the role DiHOMEs play in exacerbating ARDS, the possibly that these metabolites could serve as biomarkers for COVID-19 disease is strengthened.

Inhibition of the *in vivo* sEH can also block the toxicity of linoleate epoxides (Kodani and Hammock, 2015) through stabilizing anti-inflammatory long chain EpFAs and blocking the formation of the leukotoxin diols as demonstrated in our earlier studies (Grant et al., 1996). This evidence points to the possibility that pharmacological inhibition of the sEH will enhance and synergize with the proresolving effects of omega-3 supplementation in COVID-19 patients, leading to improvement of COVID symptoms.

This was a pilot study designed primarily to inform later experimental designs, and the relatively small sample size limits interpretation. Another limitation is that the small sample size resulted in high variability in disease severity as well as timing of disease onset and resolution which made temporal relationship between blood biomarkers and specific COVID symptoms difficult to evaluate. Our data are novel in that they shed light on a class of lipid mediators that are likely to be important for the pathogenesis of COVID-19 progression. A better understanding of mechanisms involved in COVID-19 pathophysiology are rapidly emerging, and the importance of LA and its metabolites in this disease is becoming apparent. Recent studies identified that LA binds to a fatty acid binding pocket in the SARS-CoV-2 spike protein stabilizing its confirmation in a manner that decreases viral entry into the host cell (Toelzer et al., 2020). In support of this finding, Dierckx et al. (2020) demonstrated high LA concentrations associated with lower COVID-19 severity. Neither study monitored LA metabolites therefore making it impossible to understand the biological roles of the metabolites and how they may impact interpretation from other studies. Our data described here fill a missing gap of the role bioactive mediators play in COVID-19 and emphasize a critical need to better understand the relationship between dietary lipids and their bioactive metabolites. This knowledge will bring about important insights that may lead to effective strategies to prevent rapidly worsening of COVID-19 symptoms and improved treatment efficacy. The data support further investigation on the use of DiHOME regioisomers as biological mediators or biomarkers interacting synergistically through a cross-omic network of cytokines, other lipid mediators including SPMs like resolvins, and blood chemistry to predict severe COVID-19 disease.

## Data Availability Statement

The datasets presented in this study can be found in online repositories (https://datadryad.org/stash/dataset/doi:10.25338/B8M92X). The names of the repository/repositories and accession number(s) can be found in the article/Supplementary Material.

## Ethics Statement

The studies involving human participants were reviewed and approved by University of California Institutional Review Board. The patients/participants provided their written informed consent to participate in this study.

## Author Contributions

JY, CM, and RR implement the experiments. CM, IC-P, PS, BDH, and JY wrote and revised the manuscript. BGH analyzed data. IK, PS, BGH, and JY designed the study. All authors contributed to the article and approved the submitted version.

## Conflict of Interest

BDH, CM, IC-P, and JY are partly employed by EicOsis, which is developing a potent soluble epoxide hydrolase inhibitor for pain relief. The remaining authors declare that the research was conducted in the absence of any commercial or financial relationships that could be construed as a potential conflict of interest.

## Footnotes

1 https://www.metaboanalyst.ca/
