# Supplementary material for: Plasma Linoleate Diols Are Potential Biomarkers for Severe COVID-19 Infections
Source: Front Physiol. 2021 Apr 1;12:663869. doi: 10.3389/fphys.2021.663869 (PMC8047414; doi:10.3389/fphys.2021.663869)
Supplement: Supplementary file 1 [file Table_1.docx]

**Supplementary Information for**

Plasma linoleate diols are potential biomarkers for severe of COVID-19 infections

**Cindy McReynolds^1,2^, Irene Cortes-Puch^1,2,3^, Resmi Ravindran^4^, Imran Khan^4^, Bruce G. Hammock^5^, Pei-an Betty Shih^6^, Bruce D. Hammock^1,2,7^*, Jun Yang^1,2^***

^1^Department of Entomology and Nematology, University of California, Davis, CA, USA

^2^EicOsis Human Health Inc., Subsidiary of EicOsis LLC, 1930 5th Street, Suite A, Davis, CA, ISA

^3^Division of Pulmonary, Critical Care, and Sleep Medicine, Department of Internal Medicine, University of California, Davis, CA, USA

^4^Department of Pathology and Laboratory Medicine, University of California, Davis, CA, USA

^5^Department of Veterinary Medicine, Aquatic Health, University of California, Davis, CA

^6^Department of Psychiatry, University of California, San Diego, San Diego, CA, USA

^7^UCD Comprehensive Cancer Center, University of California, Davis, CA, USA

*** Correspondence:**Jun Yang

[junyang@ucdavis.edu](mailto:junyang@ucdavis.edu)

Bruce D. Hammock

[bdhammock@ucdavis.edu](mailto:bdhammock@ucdavis.edu)

Keywords: linoleate diol_1_, lipid mediators_2_, COVID-19_3_, inflammation_4_, leukotoxin_5_, EpOME, DiHOME, ARDS

**This PDF file includes the following:**

Table 1: average cytokine levels ± standard deviation over 5-day sampling period

Figure 1: EpOME and DiHOME values from COVID-19 patients and controls and calculated EpOME: DiHOME ratios over a 5-day sampling period in COVID-19 patients compared to a single assessment in healthy controls.

**Supplementary Table 1.** Average cytokine levels ± standard deviation over 5-day sampling period in 6 COVID-19 positive patients and single timepoints in 16 healthy controls.

|  |  |  | **COVID negative** | | | **COVID positive** | | |
| --- | --- | --- | --- | --- | --- | --- | --- | --- |
| Significant |  | IP-10 | 377.51 | ± | 422.00 | 8,319.04 | ± | 13,197.36 |
|  |  | MCP-1 | 51.76 | ± | 46.99 | 198.07 | ± | 151.81 |
|  |  | IL-2R alpha | 75.96 | ± | 19.89 | 228.93 | ± | 221.50 |
|  |  | G-CSF | 32.15 | ± | 24.92 | 243.96 | ± | 185.23 |
|  |  | SCGF-b | 65,516.58 | ± | 23,927.79 | 457,401.32 | ± | 182,805.76 |
|  |  | IL-1ra | 1,464.07 | ± | 458.91 | 6,627.30 | ± | 5,657.16 |
|  |  | IL-18 | 69.87 | ± | 45.05 | 218.21 | ± | 165.27 |
|  |  | MIG | 652.77 | ± | 1,241.35 | 5,011.68 | ± | 8,872.44 |
|  |  | RANTES | 6,886.20 | ± | 9,986.27 | 82,607.91 | ± | 119,032.58 |
|  |  | IL-1 alpha | 3.64 | ± | 4.86 | 26.21 | ± | 13.29 |
|  |  | IL-1 beta | 1.73 | ± | 2.33 | 6.62 | ± | 3.10 |
|  |  | IL-2 | 13.08 | ± | 22.02 | 8.73 | ± | 3.42 |
|  |  | IL-3 | 0.01 | ± | 0.02 | 0.55 | ± | 0.44 |
|  |  | IL-4 | 1.01 | ± | 0.41 | 2.30 | ± | 0.82 |
|  |  | IL-5 | 7.78 | ± | 11.10 | 8.20 | ± | 5.06 |
|  |  | IL-6 | 0.41 | ± | 1.28 | 30.14 | ± | 18.98 |
|  |  | IL-7 | 4.41 | ± | 3.27 | 11.95 | ± | 4.90 |
|  |  | IL-8 | 4.90 | ± | 6.03 | 57.68 | ± | 38.85 |
|  |  | IL-9 | 318.33 | ± | 123.79 | 492.35 | ± | 63.57 |
|  |  | IL-10 | 9.78 | ± | 12.19 | 16.75 | ± | 5.56 |
|  |  | IL-12 (p70) | 4.45 | ± | 2.32 | 7.34 | ± | 3.13 |
|  |  | IL-12 (p40) | 46.89 | ± | 15.93 | 99.21 | ± | 58.28 |
|  |  | IL-13 | 0.91 | ± | 0.46 | 1.91 | ± | 0.55 |
|  |  | IL-16 | 94.16 | ± | 26.94 | 383.52 | ± | 258.54 |
|  |  | PDGF-BB | 611.54 | ± | 312.83 | 4,918.12 | ± | 2,593.90 |
|  |  | MIP-1b | 264.54 | ± | 97.06 | 419.52 | ± | 131.09 |
|  |  | SDF-1a | 1,657.55 | ± | 506.14 | 3,115.42 | ± | 717.18 |
|  |  | MCP-3 | 0.65 | ± | 0.88 | 25.38 | ± | 27.26 |
|  |  | LIF | 70.61 | ± | 53.20 | 77.78 | ± | 30.40 |
|  |  | IFN-a2 | 3.39 | ± | 2.01 | 9.38 | ± | 4.21 |
|  |  | IFN-g | 10.33 | ± | 9.83 | 50.09 | ± | 33.68 |
|  |  | TNF-a | 48.24 | ± | 16.31 | 128.00 | ± | 53.16 |
|  |  | TNF-b | 561.01 | ± | 223.51 | 840.87 | ± | 91.47 |
|  |  | MIF | 4,453.54 | ± | 2,413.46 | 15,533.21 | ± | 7,131.58 |
|  |  | Basic FGF | 3.70 | ± | 0.01 | 21.34 | ± | 18.93 |
|  |  | MIP-1a | 0.37 | ± | 0.56 | 8.72 | ± | 7.98 |
|  |  | M-CSF | 14.02 | ± | 5.30 | 58.77 | ± | 38.03 |
|  |  | HGF | 234.77 | ± | 61.06 | 6,463.33 | ± | 8,283.03 |
|  |  | SCF | 66.05 | ± | 12.63 | 204.00 | ± | 119.30 |
|  |  | TRAIL | 126.49 | ± | 36.31 | 222.80 | ± | 56.63 |
|  |  | CTACK | 400.80 | ± | 134.88 | 833.03 | ± | 391.99 |
|  |  | Eotaxin | 98.23 | ± | 33.87 | 147.76 | ± | 78.73 |
|  |  | VGEF | 61.21 | ± | 158.03 | 204.95 | ± | 99.50 |
| Not significant |  | GRO-a | 906.44 | ± | 222.88 | 905.37 | ± | 195.31 |
|  |  | IL-2 | 8.16 | ± | 22.02 | 7.83 | ± | 5.39 |
|  |  | IL-5 | 5.00 | ± | 11.10 | 5.00 | ± | 11.89 |
|  |  | IL-15 | 12.70 | ± | 0.00 | 12.70 | ± | 0.00 |
|  |  | IL-17 | 2.80 | ± | 0.00 | 2.80 | ± | 0.00 |
|  |  | b-NGF | 0.38 | ± | 0.00 | 0.38 | ± | 0.00 |
|  |  | GM-CSF | 0.50 | ± | 0.10 | 3.70 | ± | 0.97 |

**Supplementary Figure 1.** Individual epoxide and diol levels in patients and controls (A) and calculated EpOME: DiHOME ratios over a 5-day sampling period in COVID-19 patients compared to a single assessment in 44 healthy controls (B).

A.

B.

1. Plasma concentration (nM) of EpOME and DiHOME in five sequential samples collected from six hospitalized COVID-19 positive patients and control samples collected separately from healthy volunteers (n=44). Data from individual days is represented for each COVID patients and for each individual healthy control.
2. Ratio of EpOME: DiHOME values in healthy controls vs. hospitalized COVID-19 patients. Control values are averaged for one point ± SEM while COVID-19 patient samples are represented in chronological order of sampling after hospital admission. Lower ratios indicate that the DiHOME concentration was higher or the EpOMEs were lower and are commonly used to infer sEH activity [1]. Considering the large increase in EpOMES in COVID-19 patients compared to healthy controls, the decreased valie of the ratios in COVID-19 patients are largely driven by inceased DiHOME concentrations.

[1] D. Stefanovski, P.B. Shih, B.D. Hammock, R.M. Watanabe, and J.H. Youn, Assessment of soluble epoxide hydrolase activity in vivo: A metabolomic approach. Prostaglandins & other lipid mediators 148 (2020) 106410.
